# Trajectories: a framework for detecting temporal clinical event sequences from health data standardized to the Observational Medical Outcomes Partnership (OMOP) Common Data Model

**DOI:** 10.1093/jamiaopen/ooac021

**Published:** 2022-03-16

**Authors:** Kadri Künnapuu, Solomon Ioannou, Kadri Ligi, Raivo Kolde, Sven Laur, Jaak Vilo, Peter R Rijnbeek, Sulev Reisberg

**Affiliations:** 1 STACC, Tartu, Estonia; 2 Department of Medical Informatics, Erasmus University Medical Center, Rotterdam, the Netherlands; 3 Institute of Computer Science, University of Tartu, Tartu, Estonia; 4 Quretec, Tartu, Estonia

**Keywords:** OMOP, OHDSI, R package, observational data, trajectory

## Abstract

**Objective:**

To develop a framework for identifying temporal clinical event trajectories from Observational Medical Outcomes Partnership-formatted observational healthcare data.

**Materials and Methods:**

A 4-step framework based on significant temporal event pair detection is described and implemented as an open-source R package. It is used on a population-based Estonian dataset to first replicate a large Danish population-based study and second, to conduct a disease trajectory detection study for type 2 diabetes patients in the Estonian and Dutch databases as an example.

**Results:**

As a proof of concept, we apply the methods in the Estonian database and provide a detailed breakdown of our findings. All Estonian population-based event pairs are shown. We compare the event pairs identified from Estonia to Danish and Dutch data and discuss the causes of the differences. The overlap in the results was only 2.4%, which highlights the need for running similar studies in different populations.

**Conclusions:**

For the first time, there is a complete software package for detecting disease trajectories in health data.

## Lay Summary

Modern principles for comorbidity studies that take time dimension into account and identify temporal disease trajectories from the data were published in 2014 by Jensen et al. However, the absolute number of such studies has remained small. We believe this is because these principles were not described sufficiently to allow exact replication and have led to variations in the methods of such studies.Based on the previous publications and best practices of that field, this article proposes a 4-step framework for clinical event trajectory studies and introduces an open-source R package that implements this approach. As a proof of concept, we apply the framework for replicating the largest published trajectory study (Danish population) on Estonian population-based data. In addition, we conducted a type 2 diabetes trajectory study on Estonian and Integrated Primary Care Information (IPCI) databases from the Netherlands. To the best of our knowledge, this is the first framework for assessing disease trajectories that can be applied to any data source standardized to the Observational Medical Outcomes Partnership Common Data Model.We show that the results are highly dependent on the dataset. By comparing the results from different datasets, we highlight the opportunities and challenges of these kinds of trajectory studies. These issues have not been thoroughly described before.

## INTRODUCTION

Electronic health records are increasingly used for research. They provide a great opportunity for conducting large-scale studies of different diseases and populations that would not be feasible in classical clinical trials or cohort studies. One topic of interest in recent times has been the hypothesis-free identification of temporal disease sequences (trajectories) where one event leads to another.^1–4^ An impressive number of temporal relations have been published in various studies on whole databases^1^^,^^3^^,^^4^ and specific cohorts^5^^,^^6^ since Jensen et al^1^ published the general principles of trajectory studies in 2014. While the results provide a good characterization of these datasets, it is difficult to estimate which of these trajectories reflect local healthcare factors such as diagnosis and treatment practices unique to the local or regional healthcare system and which are generalizable globally. In order to find clinically relevant information about disease trajectories that are independent of a particular database and could potentially improve patient care, trajectory studies need to be replicated across a wider database network. The absolute number of large-scale disease trajectory studies has remained small.^4^^,^^7^ We think this is because of 2 reasons—first, there is a lack of syntactic and semantic interoperability of health data^8^^,^^9^ which makes network studies a challenge, and second, there has not been an open-source standardized implementation of an analytical framework for performing this type of analysis.

The first issue is currently being tackled by various research communities. The open-science Observational Health Data Sciences and Informatics (OHDSI) network has put a tremendous amount of effort into building an open community standard Observational Medical Outcomes Partnership (OMOP) Common Data Model (CDM). OMOP CDM uses standardized vocabulary that transforms data from disparate observational health databases into a common format. This allows the development and wider use of standardized tools for the analysis of electronic medical records regardless of the original formatting of the data.^10^ As of today, it is estimated that observational health data of 810 million distinct patients in over 330 databases are partially mapped to the OMOP model,^11^ and a wide range of studies have already been conducted on these datasets by a large OHDSI community.^12^ Using the same network for investigating temporal health event sequences would enable us to conduct trajectory studies on an unprecedented scale.

Common principles for disease trajectory studies are needed to standardize such studies. While most of the recent publications rely on the main principles published by Jensen et al^1^ in 2014 (see “Methods” section), we have found that the methods are described insufficiently for adequate replication in other datasets, making it almost impossible to verify the results or conduct a similar analysis in other settings.

In this article, we propose a standardized framework for detecting temporal clinical event trajectories in the observational health dataset, based on the previous publications and best practices of that field. It is a stepwise process starting with identifying the simplest elements of the trajectories, followed by building longer trajectories of these elements and counting the actual event sequences on that graph. We also introduce the implementation of the framework as open-source software trajectories that utilize the OMOP CDM and standardized vocabularies.

## MATERIALS AND METHODS

### Previous work

Only a few large-scale disease trajectory studies have been published so far. They mostly refer to the paper by Jensen et al^1^ published in 2014, where the general principles for modern large-scale hypotheses-free temporal trajectory analysis were described and used on a large Danish National Patient Registry. Many later studies have relied on the same dataset. For example, Siggaard et al^3^ published a browser of the results and Jørgensen and Brunak^13^ studied chronic obstructive pulmonary disease. Hu et al^2^ linked the data to the cancer registry and investigated trajectories prior to the cancer diagnosis. These principles with certain modifications have been applied to other populations as well. For instance, Han et al^5^ studied patients after depression diagnosis in UK Biobank and Paik and Kim^6^ investigated trajectories towards death in California. In 2018, Giannoula et al^4^^,^^7^ proposed a framework for detecting and clustering disease pairs in a Spanish dataset, and later extended this to include genetic information in the clustering step.

In Table 1, we have summarized the methods described in these publications. Although this is not a systematic review of trajectory studies, we believe it gives a good overview of the similarities and differences of the methods used in these works.

**Table 1.** Summary of the general principles used in recent large-scale disease trajectory studies

Publications	Jensen et al,^1^ Siggaard et al,^3^ Westergaard et al,^14^ Hu et al,^2^ Jørgensen and Brunak^13^	Giannoula et al^4^^,^^7^	Paik and Kim^6^	Han et al^5^	Our “Trajectories” framework
General principle of the trajectory analysis	First, identify event pairs, then build longer trajectories from these. In Jensen et al, cluster trajectories.	First, identify event pairs, then cluster these based on dynamic time warping	First, identify event pairs, then build longer trajectories from these and cluster these	First, identify event pairs, then build longer trajectories from these	First, identify event pairs, then build longer trajectories from these
Step 1: Study cohort	Entire Danish population, all events (whole dataset). Hu et al investigated events prior to the cancer diagnosis.	Spanish health registry (whole dataset)	Hospital deaths, events before deaths	Depression patients, events after the depression	Whole dataset or any subset of the data based on OHDSI/OMOP cohort definition principles
Step 2a: Event types in trajectories	ICD-10 level 3 diseases	ICD-9 disease diagnoses	3-Digit ICD-9-CM codes	ICD-10 level 3 diseases	Any binary condition, observation, drug era, or procedure as recorded by using OMOP vocabulary
Step 2b: Handling repeated events	Only the first event is used	Only the first event is used	Only the first event is used	Only the first event is used	Only the first event is used
Step 2c: Maximum allowed temporal distance between events	5 y	NA	1 y	NA	Any positive number of days
Step 2d: Minimum number of occurrences of event pair	10	10	NA	125 (∼0.5% of the cohort)	Any positive number or percentage of the cohort
Step 3a: Identification of significant event pairs	Sampling from matched exposed/background group by exact gender, age group, type of hospital encounter, week of the *E*1 discharge. Binomial test for association and temporal order testing. Multiple testing correction.	Matched by gender and age. Fisher’s exact test used for association testing and binomial test for assessing temporal order. Multiple testing correction.	Binomial test for association and temporal order testing. Multiple testing correction.	Matched by gender, sex, Townsend deprivation index, year of birth, year of depression diagnosis. Cox regression analysis for association testing. Binomial test for temporal order testing. Multiple testing correction.	Exposed/background group matching by using exact covariates (gender, age group and a calendar year of *E*1) and propensity score. Fisher’s exact test used for association testing and binomial test for assessing temporal order. Multiple testing correction.
Step 3b: Measurement to describe the strength of the association of event pair	Relative risk	Relative risk	Relative risk	Hazard ratio	Relative risk
Step 4: Count trajectory patterns	Allow intermediate events between trajectory elements	Allow intermediate events between trajectory elements	Allow intermediate events between trajectory elements	Not specified	Allow intermediate events between trajectory elements
Method of further clustering of the results	Clustering of the longer trajectories based on shared diagnoses	Novel clustering method based on ICD-9 hierarchy and dynamic time warping	Clustering of the longer trajectories based on shared diagnoses	Clustering based on similarity of their underlying affected systems or their etiologies	Not used
Exact software code for analysis shared	No	Partially	No	Can be requested	Yes

*Note*: In the last column, the methods used in our framework are described.

As it can be seen from the table, the first step in all studies is to identify the disease pairs and then build longer trajectories from these. Diseases in these publications use different hierarchical classification systems and are generalized to different levels. Only the first disease occurrence is taken into account. None of the studies used drugs or procedures as the trajectory events. Almost all publications used relative risk as the measurement of the strength of the disease pair. Many studies also cluster the resulting trajectories, but there is no clear agreement on the best clustering method. The visualization techniques of the results also vary across studies. None of the studies claim that identified trajectories are causal. None of the studies have published a complete ready-to-use software code either, making it difficult not only to determine exact implementation details but also to validate their methods and findings in other settings. For example, there seem to be differences in the way the group matching and clustering are conducted by different researchers, but it is hard to make an exact comparison without seeing and testing the software code. Therefore, based on these principles and our best understanding, we propose a standardized framework for future trajectory studies with the concrete implementation as a software package.

### Framework for detecting temporal health event trajectories

**Figure 1.**
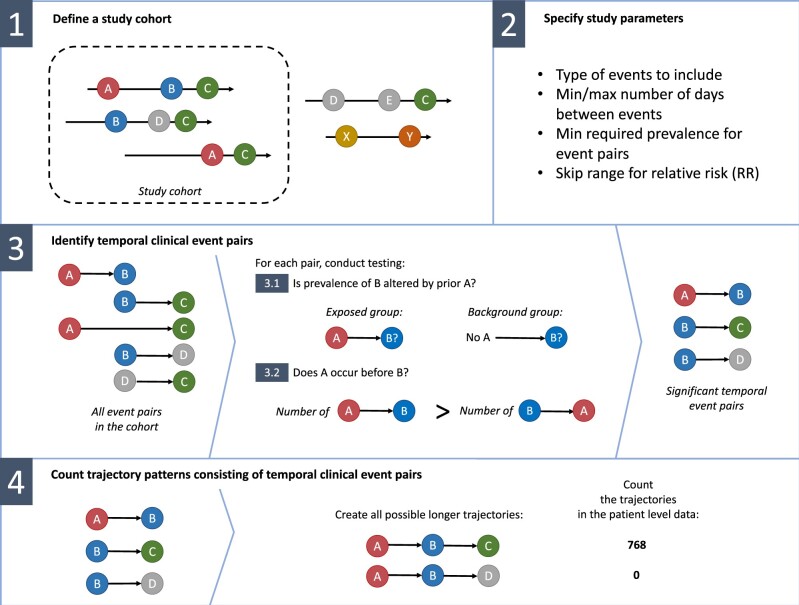
Illustration of the framework.

The proposed framework for detecting temporal health event trajectories consists of the following steps (Figure 1):

1. Define a study cohort
2. Specify study parameters
3. Identify temporal clinical event pairs
4. Count trajectories consisting of temporal clinical event pairs

### Define a study cohort

Depending on the research question, disease trajectories can be investigated either in the whole dataset or within a more specific cohort. For example, one may be interested in revealing specific treatment patterns used within a specific cohort such as type 2 diabetes (T2D), depression^5^ or deaths,^6^ ignoring clinical events related to any other patient group, or investigating trajectories separately among men and women^5^^,^^14^ or different age groups.^6^ Therefore, it is vital to clearly define the study cohort at the beginning of any disease trajectory analysis. In our framework, we use flexible and powerful cohort definition principles from OHDSI/OMOP network, where a cohort is a set of persons who satisfy one or more inclusion criteria for a duration of time.^15^ These principles have been effectively used in a number of studies across the world,^11^^,^^16^ allowing for detailed descriptions of the cohorts by using basically any kind of recorded health information. Note that a full database can also form a cohort. Identifying underlying disease pathways from a full database can discover unknown relationships in individuals and timeframes not excluded by the cohort definition.

### Specify study parameters

Within the cohort, there are many additional criteria for the trajectories that need to be specified according to the exact research question.

First, the investigator has to decide which types of clinical events are included in the analysis. While previous trajectory studies have mainly focused on diseases, we extend this approach to any event type that is recorded in observational data. Particularly, a clinical event in the context of our framework is any condition, observation, drug era, or procedure as determined by the investigator. Within OMOP CDM, all the events are coded using standardized OMOP vocabularies.

For discovering ordered temporal sequences where one event leads to another, only the first occurrence of any event for every patient within the cohort is considered, allowing us to avoid repeatedly counting the records with the same (potentially chronic) conditions. This could be a limiting factor for some studies where repetitions of the events play a role—for instance, if one is investigating the sequence of the same type of events or dynamics of numerical measurements associated to same terms. However, taking into account all occurrences of each event when conducting a study is often an impractical approach. For studying diseases that could independently occur several times (eg, seasonal influenza), shorter time frames when defining the cohort should be used, allowing the patient to be represented in the cohort with several time periods.

For each patient in the cohort, the selected events form a sequence of events. Any event along that sequence (*E*1) can be considered as a potential risk factor for future events (*E*2) where the strength of the association of the event pair can be described by relative risk (*RR*) as follows:

$$RR=\frac{\mathrm{Pr}\;(E2\;\mathrm{with}\;\mathrm{prior}\;E1)}{\mathrm{Pr}\;(E2\;\mathrm{without}\;\mathrm{prior}\;E1)}.$$

While some events increase the risk of future events (relative risk: *RR* > 1), others may decrease it (*RR* < 1). For many event pairs, the effect can be very small (*RR* close to 1) and provide little practical interest. Therefore, depending on the research question, the investigator can specify the range of relative risk of interest, leaving the event pairs that do not satisfy the *RR* range criteria out of the analysis.

Finally, there are a few parameters that can be used for fine-tuning the analysis. To focus on the most prevalent event sequences, the investigator can set a minimum prevalence for event pairs in order to include them in the analysis. Sometimes it can be useful to limit the minimum and maximum temporal distance in days between the events, preventing events that are either too close or too far apart to be included in the analysis.

### Identify temporal clinical event pairs

Although each individual sequence consists of a number of events, only a few of them have a significant effect on the following events and provide interest for the trajectory studies. The aim of this step is to identify the building blocks for creating longer trajectories. We identify event pairs where the first event tends to not only occur before the other but also alter the risk of the following event. This approach is similar to what has been used by Jensen et al^1^ and Siggaard et al.^3^

We perform extensive statistical significance testing of event co-occurrence in event sequences. First, events of each individual sequence are arranged into event pairs; every 2 events occurring in a specific order (may have intermediate events between) within a timeframe specified in the study parameters form an event pair *E*1 → *E*2. Next, for each pair that satisfies all other study parameters described in the previous section, it is assessed whether the first event alters *RR* of the following event and whether they have a significant temporal order.

For the first task, an exposed (patients having prior *E*1) and background group (matched patients from the whole cohort) are composed, and the prevalence of *E*2 in both groups is assessed. For example, Jensen et al^1^^,^^3^ matched exposed and background groups by gender, age group, type of hospital, and week of the *E*1 occurrence in the Danish dataset. The number of matching patients will quickly become very small with high levels of stratification, especially for rare events. This would require an initial database of enormous size, as was the case in Denmark.^1^ Therefore, our proposed framework requires exact matching by gender, age group and calendar year of *E*1 only. Calendar year is included to take into account underlying shifts in delivering treatment over time. Other components are combined into propensity scores and matched. Next, statistical testing is performed. The framework uses Fisher’s exact test to first assess whether the prevalence of *E*2 in the exposed group is significantly different from the background group.

For all event pairs that demonstrate a significant association, we assess whether there is a significant temporal order (direction) between *E*1 and *E*2 in the data using a binomial test (similar to Jensen et al^1^).

For both tests, the multiple testing corrected *P*-value below .05 is considered statistically significant. We propose to use the false discovery rate correction for discovery studies and the more conservative Bonferroni correction for validation studies.

### Count trajectory patterns consisting of temporal clinical event pairs

**Figure 2.**
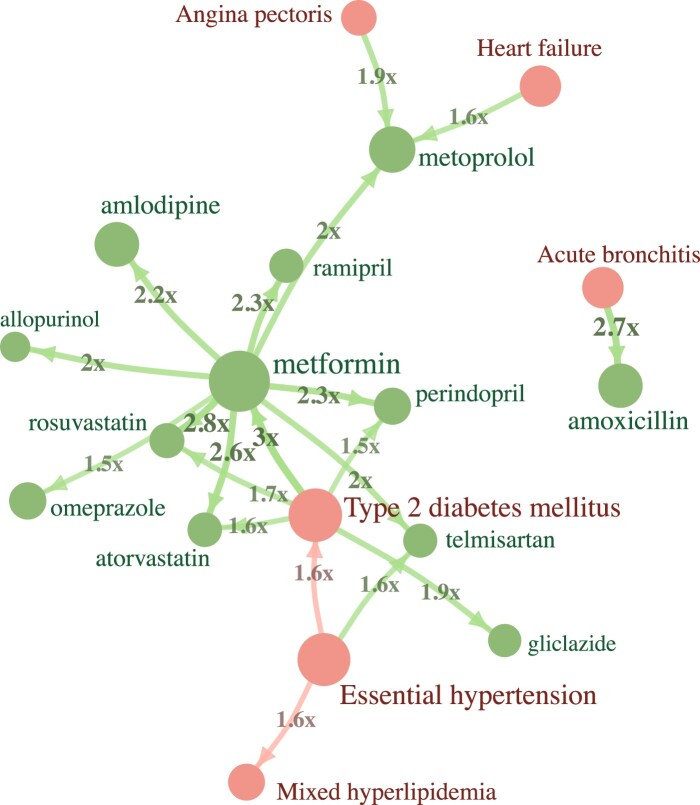
Twenty most frequent event pairs in type 2 diabetes cohort in Estonian dataset. Node size indicates the number of patients of that event record, relative risk of the future event is shown on edges. All pairs were also validated as significant in the IPCI database.

In the previous step, significant temporal event pairs—the building blocks of longer event trajectories—were identified. These can be further used to form a directed graph where each event is represented as a node and directional edges represent the temporal order between events. The graph is useful for illustrating the main trajectories within the database, especially when less frequent pairs are filtered out (Figure 2). The significant temporal event pairs are combined into all possible longer trajectories (eg, *E*1 → *E*2 and *E*2 → *E*3, producing the trajectory *E*1 → *E*2 → *E*3) and their actual occurrences are counted by evaluating them against the database. Trajectories may contain other intermediate events, the process is described in detail by Jensen et al.^1^ As a result, the list of all trajectories together with their occurrence counts is obtained. The list can be later filtered to answer questions such as how many patients have a trajectory from A to B to C.

### Implementation of the clinical event pair detection framework

The framework described above is implemented as an open-source R package freely available in GitHub,^17^ and is open for the community to add further improvements and additional features in the future. It can be run in 2 modes—either to discover event trajectories from the dataset without any prior knowledge or to validate the event pairs that were discovered in some other dataset. The only difference is that in the validation mode, the exact event pairs for testing are given as input. Detailed information on how to run the R package is described in the vignette located in the repository.

Optimal pair matching was performed using the “MatchIt” package, which calls functions from the “optmatch” package.^18^^,^^19^ For large databases, one can also use faster nearest neighbor matching.

We demonstrate the framework and the package by replicating the largest published trajectory study^3^ (Danish population) on Estonian population-based data. In addition, we conducted a T2D trajectory study on Estonian and Integrated Primary Care Information (IPCI) databases from the Netherlands. The Estonian dataset contains health data of a 10% random sample of the Estonian population (*n* = 147K patients). For each individual in the dataset, all insurance claims, digital prescriptions, and in- and outpatient electronic health records from the period 2012 to 2019 were first converted to OMOP CDM. Mortality rates in the dataset are not complete, covering approximately only two-thirds of all deaths. IPCI is a Dutch database containing the complete medical record of more than 2.8 million patients provided by more than 450 general practitioners (GPs) geographically spread over the Netherlands. In the Netherlands, all citizens are registered with a GP practice which acts as a gatekeeper in a 2-way exchange of information with secondary care. The medical records can therefore be assumed to contain all relevant medical information, including medical findings and diagnosis from secondary care. The International Classification of Primary Care (ICPC) is the coding system, but diagnoses and complaints can also be entered as free text. Prescription data contain information on product name, quantity prescribed, dosage regimens, strength, indication, and ATC codes.

## RESULTS

### Internal validation of the methods

To ensure the validity of the framework, we have equipped the package with 78 built-in tests that check various steps in the package. In addition, we designed a synthetic event pair *E*1 → *E*2 where the probability of observing *E*2 after *E*1 was 50%, and added it into random data of 1000 patients. We tested how well the package was able to detect the synthetic pair depending on selected *RR* (varying from 1.2 to 5), the count of the pair (10–100) and the count of other random events per patient (1–30). We also assessed whether the framework identifies any pair from random data without any synthetic trajectory. The corresponding test results are given in Supplementary Material. In general, the ability to detect the event pair was very good—the added event pair was successfully detected in 55 out of 62 tests. Difficulties with the trajectory detection were observed when the number of trajectories and the number of other events in the data were both small (≤20 and up to 6 additional random events per patient accordingly). In addition to true event pair, especially when the true pair occurred frequently (≥50 times), and the number of other random events was large (≥17), the framework identified other event pairs as directional (15 pairs is an extreme example). However, no directional pairs were detected in random data without the synthetic pair added, no matter how many random events were added. We also tested that if we added synthetic 3-event trajectory for 100 patients in random data (*n* = 400 patients), it was detected and counted correctly.

### Validation of Danish temporal event pairs in the Estonian population

**Figure 3.**
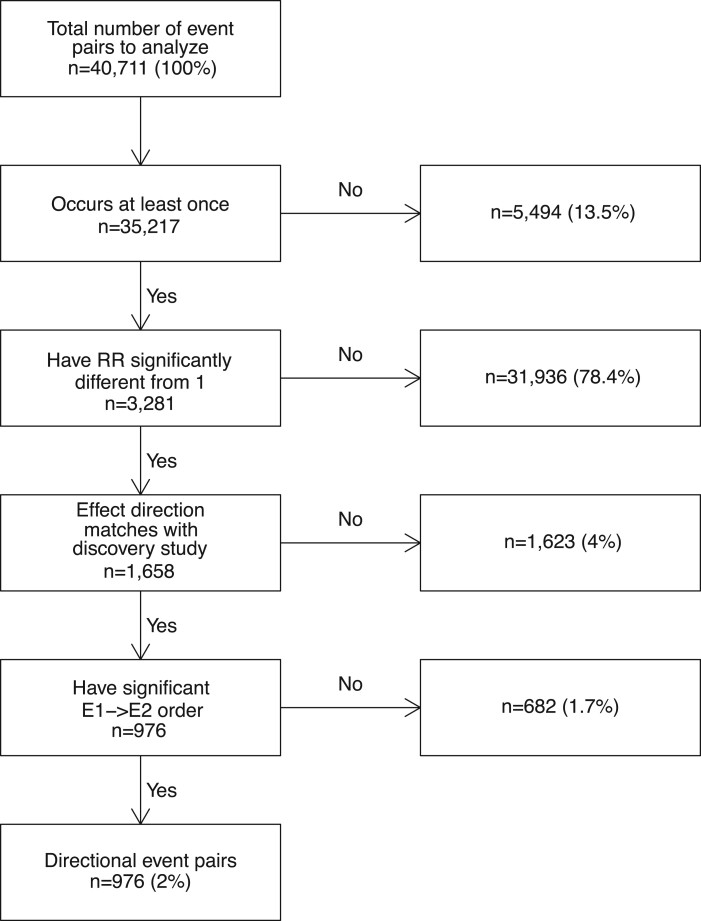
Process flow of testing Danish directional event pairs in Estonian dataset.

We analyzed 40 711 temporal event pairs reported by Siggaard et al^3^ as having significant temporal order in the Danish population and tried to confirm or reject these in Estonian data. Both datasets use The International Classification of Diseases version 10 (ICD-10) codes, the Danish dataset being much larger with 7.2 million patients and spanning 24 years (1994–2018). While the Estonian dataset contained data of all healthcare services, the Danish dataset was missing data from GPs.^2^ Here we present only the summary of the results while full details are given in Supplementary Material. Out of all pairs tested, 13.5% did not occur in Estonian data at all (Figure 3). For instance, code “K64” (hemorrhoids and perianal venous thrombosis) is one of the events in 147 temporal pairs in Denmark, but the code is never used in Estonia. There, physicians still record “I84” instead (Haemorrhoids), although the particular code was removed from ICD-10 in 2010 already.

For the majority of the pairs tested (78.4%), *RR* of the future event was not found significantly different from 1 in Estonia. However, such a high number was not a surprise as the Estonian dataset was 49× smaller in patient count and 3× in the time range. The mean counts of these nonsignificant pairs were 835 in Danish and 46 in the Estonian dataset. Differences in event frequencies play a role here. For example, code “O83” (other assisted single delivery) is frequently used in Danish data (*n* = 59 868 patients) and produced 185 significant temporal event pairs as a result (most prevalent pair “E66” overweight and obesity → “O83” occurred on 10 927 patients). However, in Estonia, the usage of “O83” is extremely rare (*n* = 12) and only 29 of the pairs tested containing that code occurred at least once, which is far from being sufficient for observing any statistical significance.

For many pairs where the preceding event altered the *RR* of the future event, the effect of the first event (increased vs decreased the risk of the second event) or the order of the 2 events was opposite of what had been reported in Denmark (4% and 1.7% of the cases respectively). All these pairs had an increased risk in the Estonian data, while a decreased effect was reported in Denmark. An extreme example is “J35” (chronic diseases of tonsils and adenoids) which had a protective effect against future “K83” (other diseases of biliary tract) in Denmark (*RR* = 0.30) but increases the same risk significantly in Estonia (*RR* = 4.5; 95% CI, 2.41–8.40).

As a result, we were able to confirm significantly altered *RR* and temporal order of 976 pairs (2.4%) (Figure 3).

### Discovering event pairs in Estonian data

**Figure 4.**
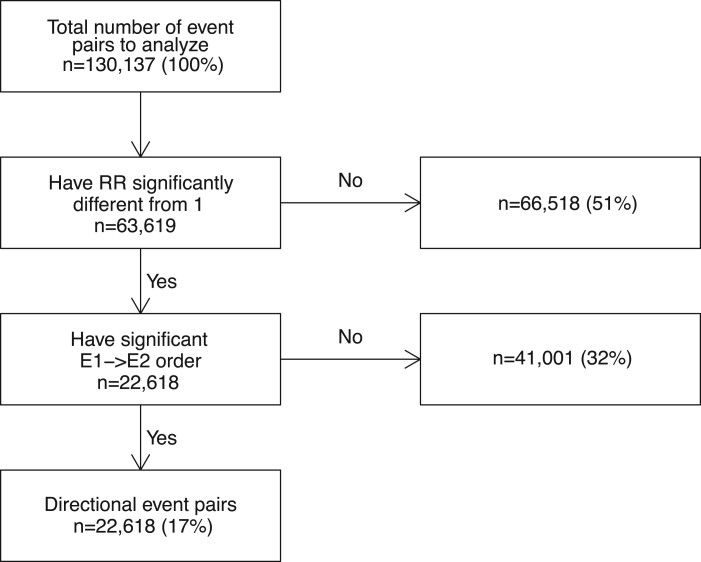
Attrition diagram of identifying directional event pairs in Estonian dataset.

To discover all temporal event pairs in Estonian data, we ran our package on the whole data without any prior knowledge of Danish findings. We used similar parameters to get comparable results (required pair count ≥20). In total, 130 137 event pairs were tested in the Estonian dataset. Out of these, 22 618 pairs in between 797 individual events were found directional and significant (Figure 4), but only 4937 pairs of them (22%) overlapped with the Danish directional pairs. What is more, for 2290 pairs (10%), the effect direction of the first event of the pair was similar to the Danish study.

**Figure 5.**
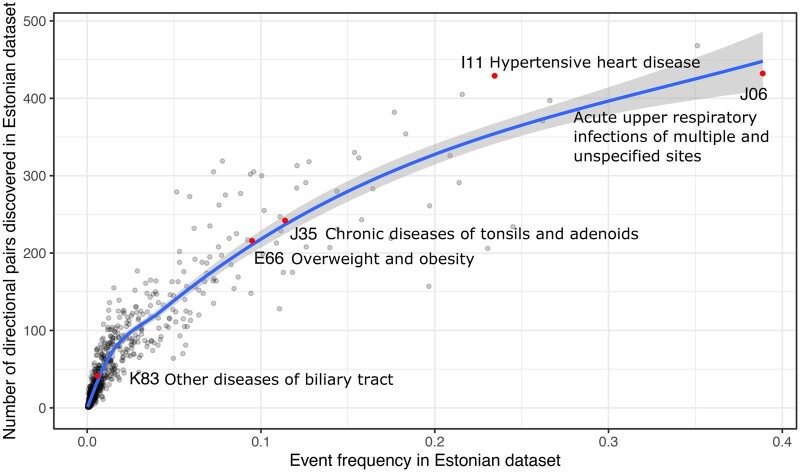
Event frequency is correlated with the number of identified significant event pairs (example on Estonian data). Diagnosis codes mentioned in this article are highlighted.

Again, differences in the frequencies of the ICD-10 codes play an important role here (Figure 5). For example, code “J06” (acute upper respiratory infections of multiple and unspecified sites) is extensively used in Estonia—recorded for 36% of the patients—leading to 432 temporal event pairs containing “J06” either as the first or the second event. In contrast, only 1.2% of Danish people have a “J06” record and just 15 temporal event pairs are found. Another example is “I11” (hypertensive heart disease) which has a frequency in Estonia 24% versus 0.5% in Denmark and temporal pair counts 425 versus 44. Such big discrepancies in individual codes may immediately affect many temporal pairs of events.

All tested and discovered event pairs are given in Supplementary Material. For privacy reasons, event counts less than 20 are hidden.

### Type 2 diabetes event trajectories in Estonia and IPCI

To illustrate a cohort-based approach, we ran a discovery study of event trajectories for T2D cohort in the Estonian database (*n* = 11 009 patients) to identify temporal event pairs that occur in at least 1% of the cohort and where the preceding event increases the risk of the second one at least 1.2 times. This particular threshold was chosen for practical reasons to eliminate event pairs where the first event increases the risk of the second event less than 20%. The discovery run on Estonian data revealed 943 significant event pairs. To validate the findings, we ran a validation study of these pairs on an independent IPCI database from the Netherlands. The validation confirmed 177 of the tested pairs (19%) while 61% of the pairs never occurred in the IPCI database, highlighting the issue of different source codes and/or OMOP CDM mappings used in these databases. In particular, IPCI uses ICPC coding system while Estonian data are based on ICD-10. The latter differentiates T2D with and without complication whereas the IPCI database has a single source code for both conditions (“Type 2 diabetes mellitus”). Therefore, pairs with “Type 2 diabetes mellitus without complication” do not occur there at all. This, of course, alters the *RR* values—in IPCI, the sole T2D diagnosis code increases the risk of future metformin 32 times (95% CI, 28.37), while in Estonia, the risk is much smaller (*RR* = 2.8) as there are other T2D diagnosis codes that precede metformin treatment.

As expected, “Type 2 diabetes mellitus” → “metformin” is the most prevalent trajectory within Estonian data as metformin is the main medication for T2D. Out of longer trajectories, “Essential hypertension” → “Type 2 diabetes mellitus” → “metformin” is the second most prevalent one. This is a somewhat expected result as high blood pressure is a previously shown risk factor for diabetes, especially when the blood pressure is uncontrolled while the order of these conditions also varies in different studies.^20–24^ The first most prevalent 3-event trajectory is “Type 2 diabetes mellitus” → “metformin” → “metoprolol,” supporting the recent findings of T2D being likely causal to hypertension.^24^ T2D damages blood vessels and increases the risk of various cardiovascular diseases,^25^ for which metoprolol was the first-line treatment until 2019 in Estonia. In the Netherlands, the most prevalent trajectory is “Cystitis” → “Urinary tract infectious disease,” supporting the previous findings that infectious diseases, including cystitis, are more prevalent among T2D patients when compared to others.^26^

All confirmed event pairs of this study, as well as the trajectories with their counts in the Estonian dataset, are given in Supplementary Material. These pairs are also available as a built-in preset in the Trajectories package so that everyone can validate those on their own database with only a few clicks. The 20 most frequent pairs are shown in Figure 2.

## DISCUSSION

In this article, we have introduced a framework and implemented an open-source software for detecting event trajectories in OMOP-formatted health data. We evaluated the framework by replicating a Danish study to identify all significant event pairs in Estonia and also conducted a T2D study on 2 different datasets.

As the results in different datasets vary considerably, it is important to understand whether the discrepancies resulted from population, the data mapping, or the framework itself. To eliminate the risk of a faulty framework, the R package is covered with 78 built-in tests, as mentioned in the “Methods” section. However, we believe that testing the whole framework in various conditions as well as testing the methods published in independent trajectory studies deserve more attention and a separate work. Due to the complexity of case–control matching, it requires a systematic approach to design good test cases and identify possible bottlenecks that are not foreseen as of today. From a population perspective, it can be seen from the results that differences in the frequencies of used event concepts (codes) play an important role in trajectory analysis. There is a strong correlation between the frequency of the event and the number of significant temporal event pairs containing that event (correlation coefficient is 0.88 in Estonia and approximately 0.99 in Denmark). Therefore, when the baseline frequencies vary in different datasets or populations, we will get different sets of significant event pairs. The reasons behind these variations in frequencies can be attributed to several factors. First, the true prevalence of the diseases can be different in different populations or datasets. Second, as we saw above, different concepts can be used in different cultures or healthcare environments to record the same underlying condition. Third, differences in source coding systems and their granularity lead to different mappings and concepts used in OMOP CDM, making them challenging to compare across several databases.^27^ If concepts were automatically generalized at a higher level, we might be able to replicate findings more effectively. This would not only resolve the problem of using different concepts but also the issue of low statistical power as the numbers in the case of generalized concepts would be higher. However, as OMOP CDM uses SNOMED Clinical Terms ontology as the underlying vocabulary, moving upwards towards the root of the ontology is a challenging task due to the multiple parent concept principle. Finding common parent concepts in various datasets would require prior analysis of these datasets. Mapping errors when transforming the original concepts to OMOP CDM vocabularies can also cause discrepancies in the event frequencies. Therefore, it is extremely important to assess the mapping quality before any trajectory study. Finally, the time span of the dataset also has its implications—the longer the observation period, the more conditions occur (such as chronic diseases or deaths, for example), increasing the frequencies of the events and leading to more event pairs with these events as a result. On the other hand, the longer the observation periods grow, the more age-dependent temporal relationships we can start observing in the data.

Another aspect that needs to be kept in mind is that event trajectories happening in the data and picked up by the software package may not be causative. For instance, confounding effects can cause spurious associations, and it is not easy to distinguish them from the causative event trajectories. The proposed framework does not yet contain any causality checks and the results characterize only the associations in the dataset. However, a temporal trend is a prerequisite for causality,^14^ and the findings could be used as hypotheses for further causality studies.

One of the weaknesses of the package is that in its current approach, it is limited to discrete or binary events only. Future extensions of the framework can also include numerical values such as laboratory measurements as events. However, as many of the measurements can vary during the same disease episode, it might become necessary to add support for repeated events into the framework, which will have a larger impact on the current principles as well.

There are a number of strengths to our approach as well. While following the principles of previously published studies, it is to our best knowledge the first open-source analysis package for investigating clinical event trajectories. Anyone can now use, examine, validate, and modify it as the source code is publicly available. It can be automatically run on any database in OMOP format, not only to characterize the data via trajectories but also to validate event pairs from other studies. The package is not limited to disease codes only as it considers other health-related events such as drug exposures, observations, and procedures, also. The whole analysis process is implemented in a single software package, making each step transparent, and as a whole, stands as a basis for reproducible science. We believe this package can greatly boost scientific studies on the analysis of temporal health events globally and will open new avenues for extending it with additional features in the future.

In the Supplementary Material, we publish event pairs and disease trajectories from the Estonian population. These can be used as comparison data for any population-level trajectory study in the future. Alternatively, we believe that there is room for improving the visualization techniques of the identified trajectories, and even without having access to any dataset, one can work on this issue by using our results.

Finally, we aimed to compare our package results to the output of another disease trajectory tool, recently published by Giannoula et al.^4^ This tool detects temporal event pairs and then clusters the trajectories using a dynamic time warping algorithm. We found the guidelines for using the tool insufficient as we were not able to run the scripts without altering them. Part of the analysis was also missing, such as the code for calculating *RR*. Attempts to contact the authors have been unsuccessful, and therefore, we were unable to compare the performance of these tools. We think this clearly illustrates why open-source pipelines are important.

## CONCLUSION

The proposed framework allows for the identification of significant clinical event progression patterns in health data standardized to the OMOP CDM. We have implemented all of this as an easy-to-use R package Trajectories that enables users to extract and visualize temporal event trajectories from OMOP-formatted observational health data and compare the results across databases.

## FUNDING

This work was supported by the Estonian Research Council (grants number PRG1095, RITA1/02-96-11); the European Union through the European Regional Development Fund (grant number EU48684); and the European Social Fund via IT Academy program. The European Health Data & Evidence Network has received funding from the Innovative Medicines Initiative 2 Joint Undertaking (JU) under grant agreement no. 806968. The JU receives support from the European Union’s Horizon 2020 research and innovation programme and EFPIA. This project has received funding from the European Union’s Horizon 2020 research and innovation programme under the Marie Skłodowska-Curie grant agreement no. 813533.

## AUTHOR CONTRIBUTIONS

KK performed formal analysis, investigation, writing—original manuscript, writing—review & editing, software, and final approval for submission. SI and KL: formal analysis, investigation, writing—original manuscript, writing—review & editing, and final approval for submission. RK and SL: methodology, writing—original manuscript, writing—review & editing, and final approval for submission. JV and PRR: methodology, writing—original manuscript, writing—review & editing, final approval for submission, and funding acquisition. SR: conceptualization, methodology, formal analysis, investigation, writing—original manuscript, writing—review & editing, software, final approval for submission, project administration, and funding acquisition.

## SUPPLEMENTARY MATERIAL

Supplementary material is available at *JAMIA Open* online.

## Supplementary Material

ooac021_Supplementary_Data

## Data Availability

The data underlying this article cannot be shared publicly for the privacy of individuals that participated in the study. The data were obtained from national health databases and can be requested via Estonian Ethics Committee (Eesti bioeetika ja inimuuringute nõukogu).
